# Smoking is associated with quantifiable differences in the human lung DNA virome and metabolome

**DOI:** 10.1186/s12931-018-0878-9

**Published:** 2018-09-12

**Authors:** Ann C. Gregory, Matthew B. Sullivan, Leopoldo N. Segal, Brian C. Keller

**Affiliations:** 1 Department of Microbiology, The Ohio State University, Columbus, OH 43210 USA; 2 Department of Civil, Environmental and Geodetic Engineering, The Ohio State University, Columbus, OH 43210 USA; 3 Division of Pulmonary, Critical Care & Sleep Medicine, New York University School of Medicine, New York, NY 10016 USA; 4 Division of Pulmonary, Critical Care & Sleep Medicine, The Ohio State University College of Medicine, 201 Davis Heart & Lung Research Institute, 473 West 12th Avenue, Columbus, OH 43210 USA

**Keywords:** Bacteriome, Lung, Virus, Virome, Microbiome, Metabolome, Smoking

## Abstract

**Background:**

The role of commensal viruses in humans is poorly understood, and the impact of the virome on lung health and smoking-related disease is particularly understudied.

**Methods:**

Genetic material from acellular bronchoalveolar lavage fluid was sequenced to identify and quantify viral members of the lower respiratory tract which were compared against concurrent bronchoalveolar lavage bacterial, metabolite, cytokine and cellular profiles, and clinical data. Twenty smoker and 10 nonsmoker participants with no significant comorbidities were studied.

**Results:**

Viruses that infect bacteria (phages) represented the vast majority of viruses in the lung. Though bacterial communities were statistically indistinguishable across smokers and nonsmokers as observed in previous studies, lung viromes and metabolic profiles were significantly different between groups. Statistical analyses revealed that changes in viral communities correlate most with changes in levels of arachidonic acid and IL-8, both potentially relevant for chronic obstructive pulmonary disease (COPD) pathogenesis based on prior studies.

**Conclusions:**

Our assessment of human lung DNA viral communities reveals that commensal viruses are present in the lower respiratory tract and differ between smokers and nonsmokers. The associations between viral populations and local immune and metabolic tone suggest a significant role for virome-host interaction in smoking related lung disease.

**Electronic supplementary material:**

The online version of this article (10.1186/s12931-018-0878-9) contains supplementary material, which is available to authorized users.

## Background

Smoking is the leading cause of chronic obstructive pulmonary disease (COPD) and the third highest cause of death globally [1, 2]. Despite the clear associated risk, only a fraction of smokers eventually develop COPD [2, 3]. What causes some smokers, and not others, to develop COPD remains unknown and an area of active research [2–5]. Recent work examining the lung bacteriome of individuals with moderate to severe COPD revealed decreased bacterial diversity compared to nonsmokers [6–11]. As a result, it has been proposed that changes in lung-resident bacterial communities may lead to COPD [4–8]. However, respiratory tract bacterial communities of individuals with mild COPD, “healthy” smokers, and nonsmokers are not significantly different [8, 11–13], suggesting that factors other than commensal bacteria may trigger COPD development.

To date, few studies have examined lung viral communities where the vast majority of viruses have been identified as bacteriophages [14–18]. Phages impact bacterial communities through direct and indirect interactions. Though phage ecological roles are unknown in the lung, their activities are relatively well-documented in the oceans where they regulate bacterial population sizes, diversity, metabolic outputs, and gene flow [19–24]. In humans, phages may stimulate the immune system leading to immune-mediated microbial competition [25], tax the immune system enabling opportunistic infection [26], or work symbiotically at human mucosal surfaces providing a source of additional immunity [27]. Thus, changing lung viral communities could alter the bacteriome leading to dysbiosis and disease progression in pre-affected (e.g., COPD) individuals [6–8]. Here we utilized a historical cohort to explore the impact of smoking on the lung microenvironment with specific focus on the role of double-stranded DNA (dsDNA) viruses. To do this, we applied a quantitative sample-to-sequence dsDNA viral metagenomic processing pipeline [28] that maintains relative abundances between samples and used these data as a baseline to compare and ecologically contextualize lung viromes in relation to lung bacteriomes, metabolomes, and immunologic profiles of “healthy” smokers and nonsmokers.

## Methods

### Sample collection and processing

Between 2010 and 2013, bronchoalveolar lavage (BAL) fluid was collected from 30 asymptomatic subjects (10 nonsmokers, 14 former smokers, and 6 current smokers) as part of previous studies evaluating the lower airway bacteriome and inflammation [29, 30]. Briefly, bronchoscopy was performed via nasal approach and avoiding suctioning until the scope was positioned for sampling. Sequential BAL was collected from the lingula and right middle lobe, combined, and processed. Metabolites and cytokine numbers were measured as previously described [29, 30], and identified metabolites were reported if present in ≥50% of the samples. Intensity data were mean-centered and divided by the standard deviation using MetaboAnalyst [31]. For in vivo cytokines, 39 cytokines were measured with a Luminex 200IS (Luminext Corp, Austin, TX) using Human Cytokine Panel I (Millipore, Billerica, MA). Data were analyzed with MasterPlex TM QT software (version 1–2, MiraiBio, Inc. Alameda, CA).

### 16S rRNA gene sequencing

The 16S rRNA gene sequencing dataset collected as part of [30] was analyzed in the context of smoking status. The creation of this dataset has been previously described [30]. Briefly, acellular BAL was obtained after centrifugation at 500 x *g* for 10 min at 4 °C followed by DNA extraction via ion exchange column (Qiagen). Additionally, DNA was extracted from pre-bronchoscopy saline to determine the level of background microbial contamination. The V4 region of the bacterial 16S rRNA gene was amplified in duplicate reactions, using primer set 515F/806R, which nearly universally amplifies bacterial and archaeal 16S rRNA genes [32, 33]. Each unique barcoded amplicon was generated in pairs of 25 μl reactions with the following reaction conditions: 11 μl Polymerase Chain Reaction (PCR)-grade H2O, 10 μl Hot Master Mix (5 Prime Cat# 2200410), 2 μl of forward and reverse barcoded primer (5 μM) and 2 μl template DNA. Reactions were run on a C1000 Touch Thermal Cycler (Bio-Rad) with the following cycling conditions: initial denaturing at 94 °C for 3 min followed by 35 cycles of denaturation at 94 °C for 45 s, annealing at 58 °C for 1 min, and extension at 72 °C for 90 s, with a final extension of 10 min at 72 °C. 16S rRNA gene amplicons were sequenced with Illumina MiSeq and analyzed using QIIME. Using this dataset, we normalized absolute operational taxonomic unit (OTU) sequence counts to obtain the relative abundances of the microbiota in each sample. These relative abundances at 97% OTU similarity and each of the 5 higher taxonomic levels (phylum, class, order, family, genus) were tested for univariate associations with clinical variables. The ade4 package in R was used to construct Principal Coordinate Analysis (PCoA) based on weighted UniFrac distances [34, 35].

### Shotgun sequencing

DNA extracted from the same acellular BAL samples described above was sheared with a Covaris E210 Focused-ultrasonicator. Libraries were constructed with the NEBNext Ultra DNA Library Prep Kit for Illumina (New England Biolabs, Ipswich, MA) and sequenced with Illumina MiSeq. Reads were QC’d and trimmed using BBDuk (BBtools package) [36], de-duplicated, and aligned to the human genome (95% identity) with BBMap [36]. Following processing, each virome had on average > 1 million reads (Additional file 1: Table S1). Cross-assembly of all 30 viromes using SPAdes [37] assembled no viral contigs > 500 bp. Consequently, to determine if viruses were present in a sample, reads were aligned using Bowtie2 [38] to a custom viral database composed of Viral RefSeq release 78, the VirSorter database [39], 23 core gut phages [36–40], and the crAssphage genome (GenBank Accession #JQ995537). Viruses with reads aligned at ≥95% percent identity [41, 42] to a consecutive 200 bp stretch of the genome were considered present in the lung virome. Median coverage was normalized to decontaminated virome read numbers to determine viral relative abundances. While 16S rRNA data was available from saline control samples from earlier studies [29, 30], insufficient amounts of saline and oral rinse control specimens remained for repeat testing by shotgun sequencing.

### Statistics

Ecological diversity statistics were performed using vegan in R [43]. Statistical outliers were evaluated using “pcout” in the mvoutlier package [44]. Bray-Curtis distances were calculated with and without outliers and were statistically ordinated using PCoA; bivariate ellipses were fit to the ordination using “ordiellipse” based on smoking status, race, and gender, and centroids were assessed to be significantly different using the “envfit” functions in vegan. Mantel’s tests using a spearman correlation were used to correlate viral Bray-Curtis distances. Differentially abundant viral populations across smokers and nonsmokers were determined with Metastats [45, 46]. For metabolic data, bacterial and viral abundances were vector-fit to the PCoA (“envfit” function). A total of 9999 permutations were used for all vector and centroid fitting, and Mantel’s tests were used to further confirm the correlations between changes in metabolic data and changes in bacterial and viral abundances. These vector fittings and Mantel’s test *p*-values were Bonferroni-corrected. To determine if viral pneumotypes existed, the SPIEC-EASI package [47] was applied using the Meinshausen and Bühlmann (MB) method to infer associations between viral populations. A batch file of all bioinformatics parameters and code can be found on iVirus in Cyverse (/iplant/shared/iVirus/Lung_Virome).

## Results

### Cohort

In a previous study, we explored the association between the lower airway bacteriome and inflammation in healthy, asymptomatic individuals. Utilizing this historical cohort [30], we selected 30 subjects (20 current or former smokers and 10 nonsmokers, Table 1) for which sufficient BAL sample remained for additional virome analysis to analyze the relationship between smoking and the lower airway microenvironment. As previously described [29], nonsmokers were enrolled from the NYU CTSI-sponsored Healthy Volunteers Bronchoscopy Cohort, characterized by subjects with no significant smoking history, normal spirometry, and absence of pulmonary, cardiovascular, renal, or endocrine disease. Smokers were enrolled from the NYU Early Detection Research Network (EDRN, 5U01CA086137–13), a longitudinal cohort consisting of approximately 2000 subjects with substantial smoking history (43.8 ± 24.3 pack-years). Smoking status was obtained during clinical interview screenings. Smokers and nonsmokers were similar in height, weight and gender distribution, whereas older, white participants were over-represented among smokers. In terms of lung function, smokers and nonsmokers had normal forced vital capacity (FVC), forced expiratory volume in 1 s (FEV_1_), and diffusing capacity of the lungs for carbon monoxide (DLCO), whereas smokers had lower mean FEV_1_/FVC ratios.

**Table 1:** Participant characteristics

Demographics	Smokers (*n* = 20)	Nonsmokers (*n* = 10)	Statistical Differences
Age, yr	63.7 (58.5–67.2)	36.2 (28.3–41.9)	*p < 0.00001*
Gender			*p* < 0.3980^b^
Male	13 (65%)	8 (80%)	
Female	7 (35%)	2 (20%)	
Height, cm	172.7 (165.1–179.1)	176.5 (173.4–181.6)	*p* < 0.3125
Weight, kg	79.8 (69.4–91.6)	85.5 (75.1–101.2)	*p* < 0.6560
Race			*p < 0.0037* ^b^
White	19 (95%)	5 (50%)	
Other	1 (5%)	5 (50%)	
Pulmonary Function Testing^a^			
FVC (% Predicted)	96.9 (90.2–102.8)	96.0 (90.0–103.7)	*p* < 0.8337
FEV_1_ (% Predicted)	94.1 (86.7–104.2)	97.1 (86.2–103.4)	*p* < 0.9840
FEV_1_/FVC	71.7 (69.9–78.4)	83.5 (77.7–84.3)	*p < 0.0220*
DLCO (% Predicted)	91.0 (79.3–99.0)	94.0 (82.0–98.0)	*p* < 0.4593
BAL Immune Cell Counts (%)			
Macrophages	90.8 (87.8–94.8)	90.8 (86.0–93.5)	*p* < 0.9840
Lymphocytes	6.2 (3.9–8.7)	6.6 (5.1–11.5)	*p* < 0.5552
Neutrophils	1.8 (1.3–2.9)	1.3 (1.2–1.6)	*p* < 0.1645
Eosinophils	0.3 (0.1–0.6)	0.2 (0.1–0.3)	*p* < 0.2670

Data is given in counts or median values and interquartile ranges. All comparisons are Mann-Whitney U-test results unless noted. ^a^Data based on NHANES predicted values. ^b^Chi-squared analysis

### Composition of the lung Virome

**Fig. 1:**
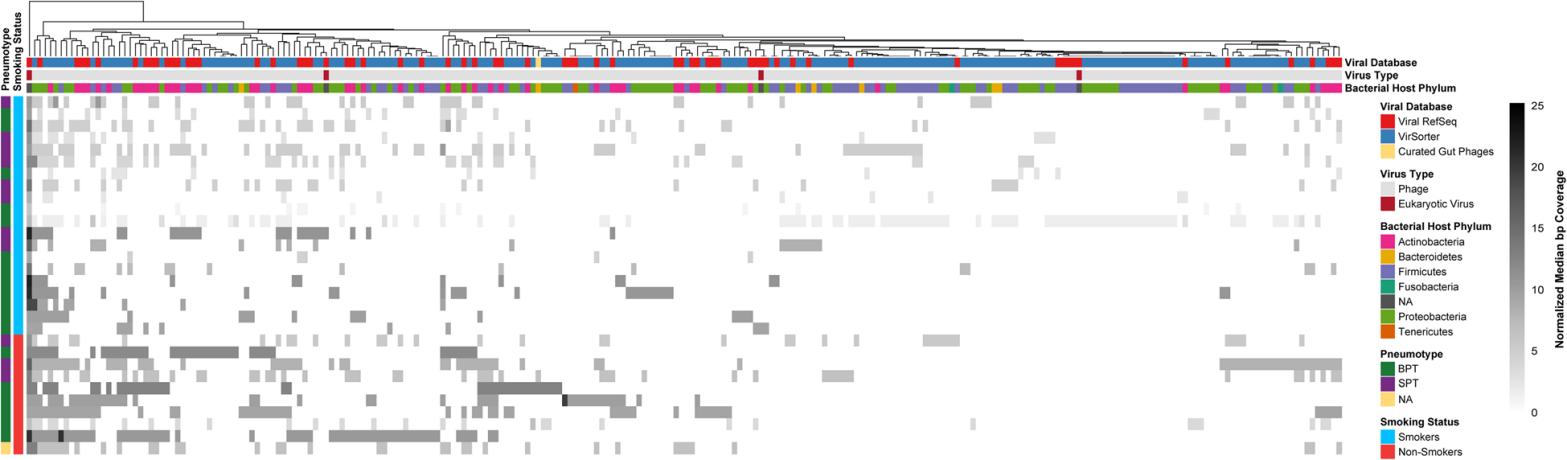
Identity and relative abundances of viruses in the smoker and nonsmoker lung. Heatmap of relative abundances of the 247 viral populations based on median normalized coverage for each virome. Each row shows the viral community composition of smokers and nonsmokers, also identified by bacterial pneumotype as determined in [26, 27]. Each column represents a distinct viral population coded by host phylum, virus type, and database in which the viral genome can be found. The dendrogram above the heatmap shows hierarchical clustering of viral populations based on abundances across the different viral communities. BPT = background predominant taxa, SPT = supraglottic predominant taxa, NA = not assessed

DNA was extracted from acellular BAL and sequenced with Illumina MiSeq. Despite removing reads mapping to the human genome at > 95% identity, many contaminating human reads remained. Of the almost 35 million reads following human decontamination across all 30 samples, only 9730 reads (0.03% of total reads) mapped to our curated viral database (Additional file 1: Table S1). In total, these reads mapped to 247 different viral populations (Fig. 1). All but one of the viruses detected were found in the Viral RefSeq or VirSorter [39] databases. One virus classified as a core gut virus [40] was detected in the lung of two individuals.

Only three eukaryotic DNA viruses were detected in the acellular BAL samples (Fig. 1). These included human herpesvirus 8, human adenovirus 2, and human papillomavirus type 4. All eukaryotic viruses were present in only one or two subject’s lung viromes.

Similar to previous findings [14–17], the majority of lung viruses (> 85% of mean viral community abundances) identified in our study were bacteriophages. The identified phages are predicted to infect a broad array of bacterial phyla based on the hosts of reference viruses in Viral RefSeq and VirSorter [39] with 37% infecting Proteobacteria, 36% Firmicutes, 23% Actinobacteria, 3% Bacteriodetes, 1% Fusobacteria, and < 1% Tenericutes (Additional file 2: Figure S1A). Of the Proteobacteria hosts, the majority included *Neisseria*, *Escherichia*, *Acinetobacter,* and *Burkholderia* (Additional file 2: Figure S1B). Among the Firmicutes and Actinobacteria hosts, the majority belong to a single genus, with 60% from the genus *Streptococcus* and 78% from the genus *Propionibacterium*, respectively (Additional file 2: Figure S1C, D). All of the Bacteriodetes hosts that could be annotated (5 out of 6) belonged to the genus *Prevotella,* while *Leptotrichia* and *Spiroplasma* were the only genera identified from the phyla Fusobacteria and Tenericutes, respectively.

Phage abundances were summed based on host genera across all 30 lung viromes to create the total virome. Based on percentages of the total virome, *Propionibacterium* phages were the most abundant across the 30 lung viromes, making up 29% of the total viral community (Additional file 3: Figure S2). The next most abundant phages were *Streptococcus, Burkholderia, Escherichia,* and *Bacillus* phages, each making up > 10% of the mean viral community (Additional file 3: Figure S2). Lastly, phages infecting the genera *Acinetobacter, Neisseria, Mannheimia, Staphylococcus, Gardnerella, and Shigella* made up > 2% and phages infecting the genera *Bartonella, Lactobacillus, Methylbacterium, Salmonella, Streptomyces, Prevotella, Veillonella, and Eubacterium* made up > 1% of total viral community (Additional file 3: Figure S2).

### Absence of viral Pneumotypes

Previous work in the human gut identified three distinct microbial enterotypes based on co-occurrence of microbial populations and predominance of specific microbial groups [48]. Using the same samples as used in the current study, we previously identified lower respiratory tract bacterial pneumotypes through hierarchical clustering and PCoA analysis of bacterial communities based on 16S rRNA abundances [29, 30]. Bacterial pneumotypes were present irrespective of smoking status. Similarly, we used hierarchical clustering of viral population abundances to evaluate for viral pneumotypes (Fig. 1; hierarchical clustering of viral communities by individual subject not shown) but found no clear clusters. To further assess if viral pneumotypes were present in our samples, we used SPIEC-EASI which forms a co-occurrence network based on correlations between viral populations (Additional file 4: Figure S3). If distinct viral pneumotypes existed across our samples, we should see clear separation of viral populations into clustered groups. We thus conclude that we could not find distinct viral pneumotypes in our cohort.

### Lung Virome comparisons between smokers and nonsmokers

**Fig. 2:**
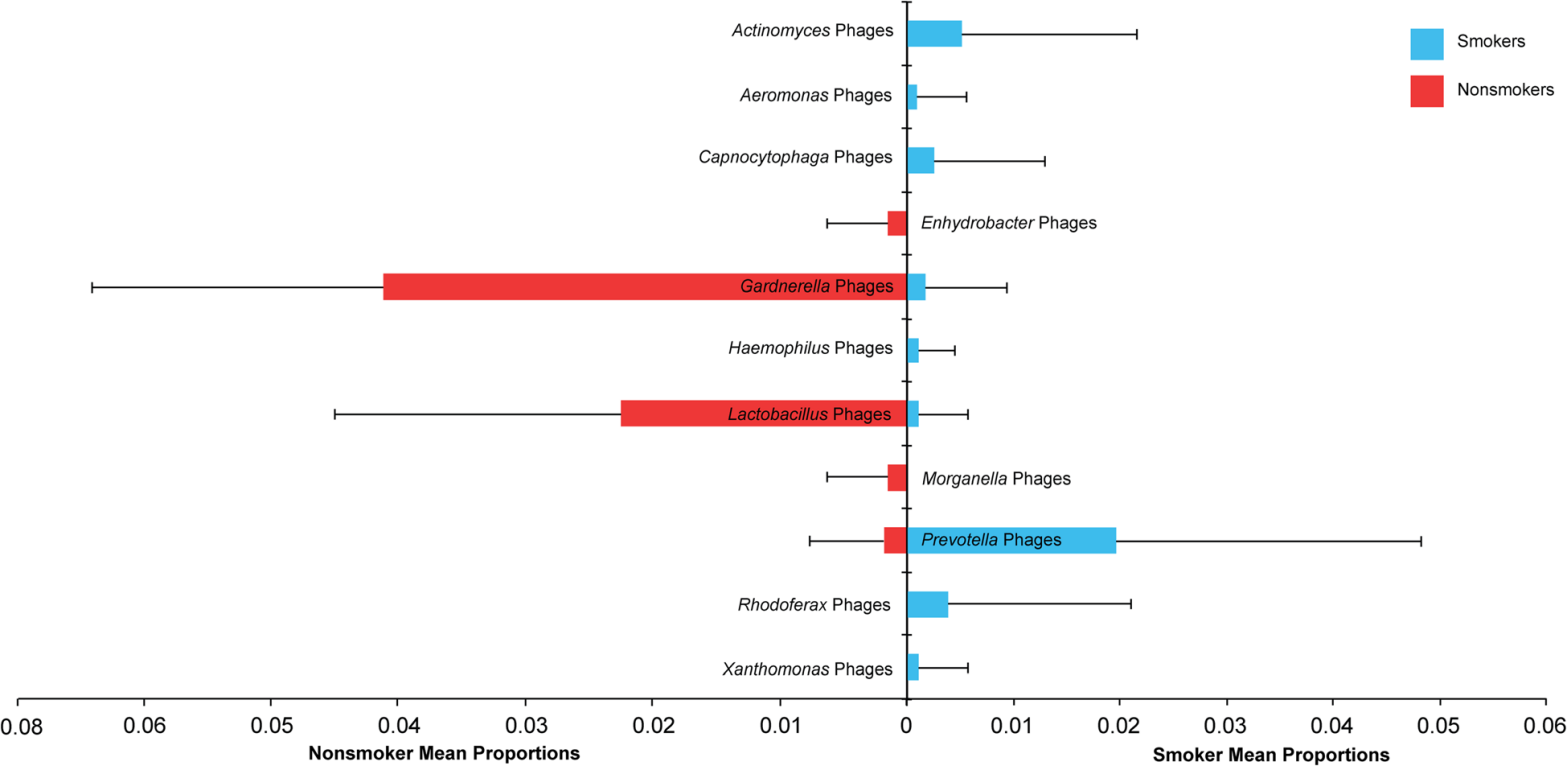
Differentially abundant phage types between smokers and nonsmokers. All statistically significant (Metastats, 1000 permutations, *p* < 0.05) phage differences based on changes in relative abundances are shown

We next assessed lung virome composition by smoking status. While a large fraction of the viral populations detected across the 30 samples were shared between smokers and nonsmokers (29%), there were clear differences between abundances of certain phage groups in smoker and nonsmoker viromes. *Prevotella* phages were at least two-fold higher in the smoker virome, whereas in the nonsmoker virome, *Lactobacillus* and *Gardnerella* phages were 10-fold more abundant. Across individuals, statistical analyses of differentially abundant viral populations using Metastats [45, 46], a tool designed to handle sparse counts, revealed similar results. *Prevotella* phages (Metastats: *p* = 0.02) were significantly increased among smokers while *Lactobacillus* and *Gardnerella* phages (Metastats: *p* = 0.001, both) were significantly increased among nonsmokers (Fig. 2). Furthermore, phages infecting *Actinomyces*, *Aeromonas*, *Capnocytophaga, Haemophilus, Rodoferax,* and *Xanthomonas* were also increased among smokers, and phages infecting *Enhydrobacter* and *Morganella* were increased among nonsmokers (Metastats: *p* < 0.05).

Some rare viral populations were unique to smoker or nonsmoker total viral communities (Additional file 5: Figure S4). For example, *Actinomyces, Capnocytophaga*, *Haemophilus* and *Rhodoferax* phages were found only in smokers, and *Enhydrobacter*, *Enterobacter, Holospora, Morganella,* and *Spiroplasma* phages were found only in nonsmokers. Eukaryotic DNA viruses were only found in the lungs of smokers (Additional file 5: Figure S4).

### Ecological comparisons between smokers and nonsmokers

**Fig. 3:**
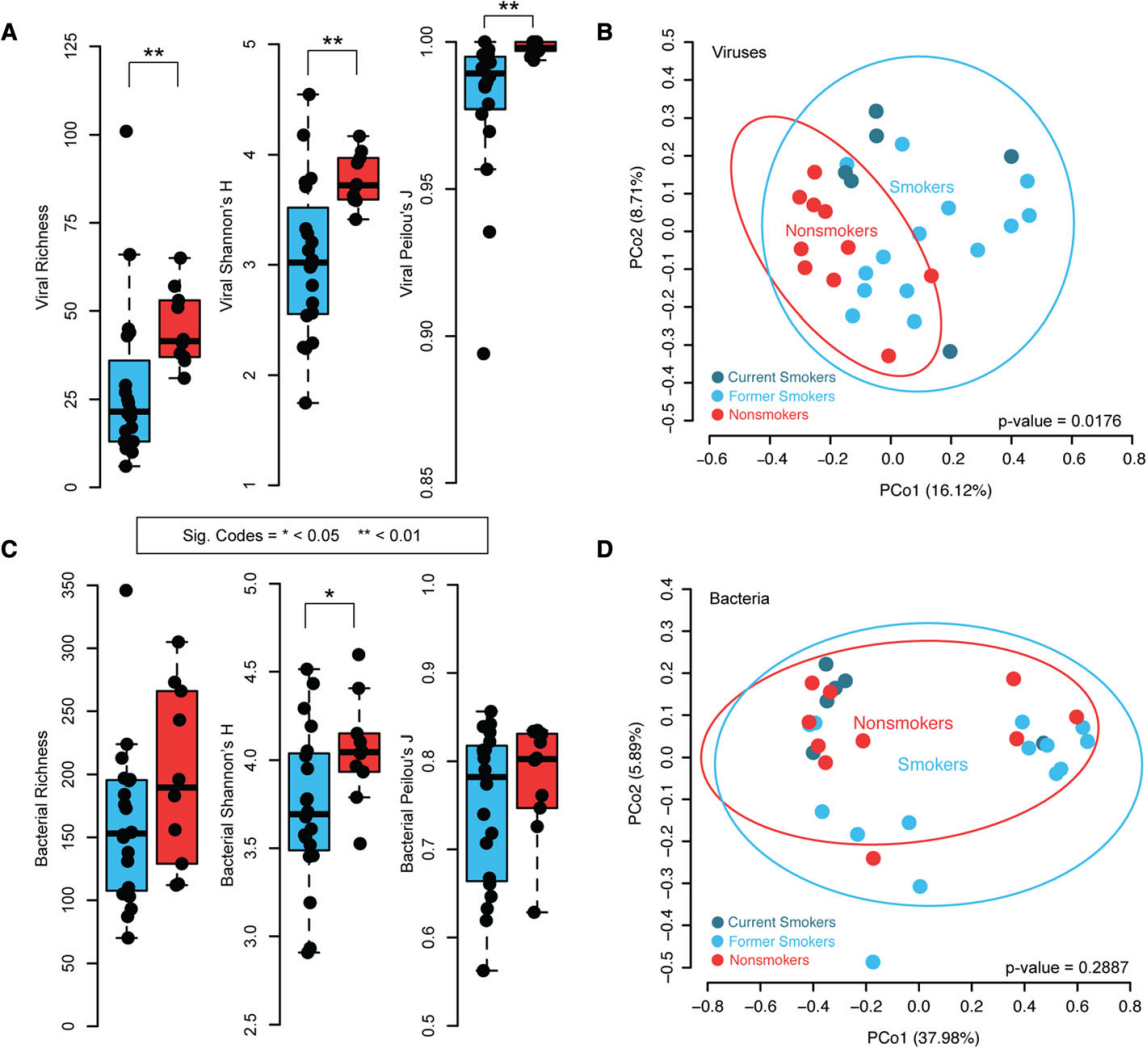
Biodiversity Metrics for Viruses & Bacteria. **a**, **c** Richness, diversity (Shannon’s H), and evenness (Peilou’s J) of smoker (blue bars) and nonsmoker (red bars) viral and bacterial communities, respectively. **b**, **d** PCoA of Bray-Curtis distances between viral and bacterial communities, respectively. Smoking status was factor fit to the PCoA plot, with blue and red ellipses represents smoking and nonsmoking statuses, respectively

We next examined the lung virome ecology of smokers and nonsmokers. Ecological α diversity measures of richness, biodiversity (Shannon’s H), and evenness (Peilou’s J) (Fig. 3a) were significantly different (Mann-Whitney U-test; *p* < 0.01) between smoker and nonsmoker viromes with smokers exhibiting lower values in all analyzed metrics. Further, viral community structure (β diversity) was significantly fit by smoking status (Fig. 3b, Bray-Curtis distances, bivariate ellipse fitting (BEF): *r*^2^ ≥ 0.32, *p* ≤ 0.02). Because some effects of smoking are reversible upon cessation, we performed a subgroup analysis of viral communities from current and former smokers and found no significant virome differences (BEF: *p* = 1.00). We also tested whether viral communities could be fit based on their paired bacterial pneumotypes [29, 30] and found no significant association between viral communities and bacterial pneumotypes (BEF: *r*^2^ ≥ 0.17, *p* ≤ 0.14). Finally, we tested if, within smoker and nonsmoker viral communities, there was significant fitting based on their paired bacterial pneumotype and again found no significant fitting (BEF: within smoker: *r*^2^ ≥ 0.12, *p* ≤ 0.10; within nonsmoker: *r*^2^ ≥ 0.34, *p* ≤ 0.20).

Since differences in age and race were noted among the smoker and nonsmoker groups, we tested whether these variables affect the β diversity distribution of the samples. Age was not significantly correlated to Bray-Curtis bacterial and viral community distances (Mantel’s test; bacteria: *r* = − 0.04, *p* < 0.71; virus: *r* = − 0.001, *p* < 0.46). Race also did not significantly explain the variance across all 30 bacterial or viral communities (BEF: bacterial: *r*^2^ ≥ 0.08, *p* ≤ 0.74; viral: *r*^2^ ≥ 0.08, *p* ≤ 0.61 for race).

Previous studies demonstrated changes in the lung bacteriome in moderate to severe COPD [7, 13], but no differences were found in lung bacterial community structure in healthy smokers without COPD compared to nonsmokers [12]. Consistent with this, and in contrast to the lung virome, we found no significant differences in bacterial α diversity (richness, Mann-Whitney U-test, *p* < 0.15; evenness, Peilou’s J: Mann-Whitney U-test, *p* < 0.50) and only a slight difference based on Shannon index (Mann-Whitney U-test; *p* < 0.05) (Fig. 3c). Differences in bacterial β diversity were noted, but these differences were not explained by smoking status (Fig. 3d, BEF: *r*^2^ ≥ 0.01, *p* ≤ 0.67). Instead, bacterial communities in our study were previously found to separate based on pneumotypes [29, 30]. Given these results, it was not surprising that bacterial and viral Bray-Curtis distances did not correlate (Mantel’s *r* = 0.09, *p* < 0.06).

Low biomass specimens, such as BAL fluid, are at risk of confounding from environmental contamination [49]. To address this, we examined bacteriome differences between pre-bronchoscopy control saline samples from smokers and nonsmokers and found no significant differences (Additional file 6: Figure S5). No *Propionibacterium* bacteria, common reagent and laboratory contaminants, were detectable within the background. In a subgroup of subjects, we previously demonstrated a lack of upper airway carryover into these lower airways specimens (reported in Fig. 2 of [29]).

### Metabolic differences between smokers and nonsmokers

**Fig. 4:**
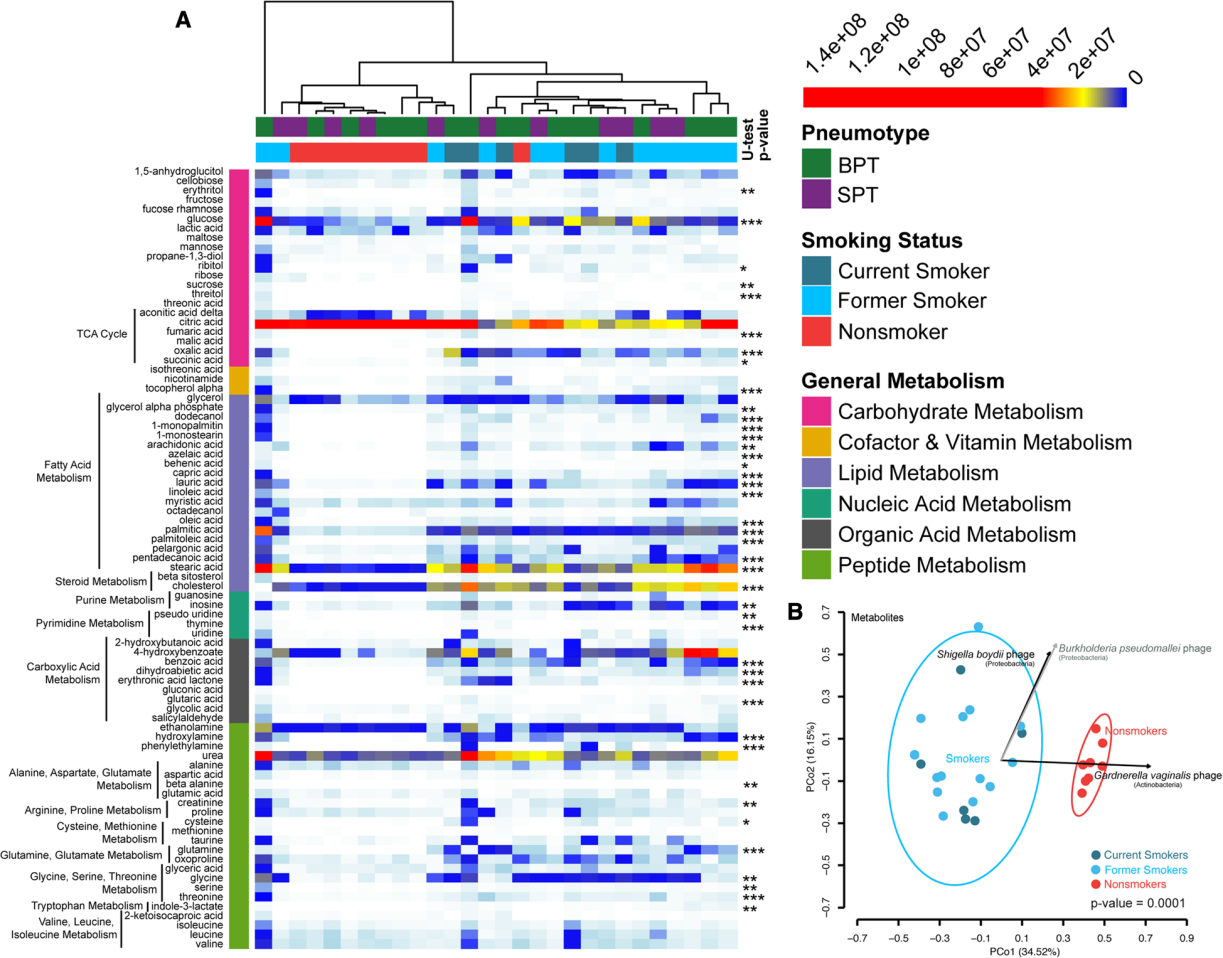
Comparison of smoker and nonsmoker BAL metabolites. **a** Heatmap of examined metabolites in BAL fluid. Each row shows the ion intensity for a specific metabolite. Metabolites are grouped based on metabolic pathways. Each column shows the BAL fluid metabolic profiles of smokers and nonsmokers, also identified by bacterial pneumotype as determined in [25, 26]. Progression from white to blue to yellow to red indicate increased metabolite content. Asterisks indicate significantly different metabolites between smokers and nonsmokers as assessed by Bonferroni corrected Mann-Whitney U-test (* = *p* < 0.05, ** = *p* < 0.01, *** = *p* < 0.001) (**b**) PCoA of Bray-Curtis distances between different metabolic profiles. Smoking status and bacterial and viral abundances were factor and vector fit to the PCoA plot, respectively. Blue and red ellipses represent factor fitting of smoking and nonsmoking status, respectively. Black vector arrows denote significant vector fitting of bacterial and viral populations based on 9999 permutations and following Bonferroni correction (*p* < 0.05). The gray vector arrows denote significant vector fitting of bacterial and viral populations based on 9999 permutations and significant Mantel test results following Bonferroni correction (*p* < 0.05). BPT = background predominant taxa, SPT = supraglottic predominant taxa

To assess the impact of smoking on cellular activities at the functional level, we compared the lung BAL metabolomes of smokers and nonsmokers. In total, we identified 83 distinct metabolites and assessed their abundances across individual smokers and nonsmokers (Fig. 4a). Most metabolites were significantly different between smokers and nonsmokers (Bonferroni corrected Mann-Whitney U-test, *p* < 0.05). These included metabolites involved in multiple metabolic pathways; among the top differences, fatty acid and carboxylic acid metabolites were significantly elevated in smokers.

Hierarchical clustering by metabolic profile showed strong clustering of nonsmokers, with nonsmokers having lower metabolite levels than smokers for all metabolites except citric acid. Smoker metabolic profiles also clustered, but with greater variation (Fig. 4a). Metabolic profile Bray-Curtis distances supported the hierarchical clustering and demonstrated significant fitting by smoking status, with low variance among nonsmokers and more variance among smokers (Fig. 4b, BEF: *r*^2^ ≥ 0.56, *p* ≤ 0.0001).

We next evaluated whether distinct bacterial or viral populations may be associated with metabolic profile differences by vector fitting all bacterial and viral abundances to the metabolite Bray-Curtis distances (Fig. 4b). Because PCoA are non-planar, we also ran regressions between Bray-Curtis distances of the bacterial and viral population abundances and the metabolite data converted into Euclidean distances using Mantel’s tests. Following Bonferroni correction, three populations emerged as significantly associated with metabolic profile differences (Fig. 4b, *p* < 0.05); all three populations were viruses. Surprisingly, no changes in bacterial abundances were significantly associated with metabolic differences between smokers and nonsmokers. Changes in the abundances of the Proteobacteria phages, *Shigella boydii* phage and *Burkholderia pseudomallei* phage, were associated with a metabolic shift towards smokers, while an Actinobacteria phage, *Gardnerella vaginalis* phage, appeared to influence metabolic differences in nonsmokers.

### Associations between viruses and the pulmonary environment

**Fig. 5:**
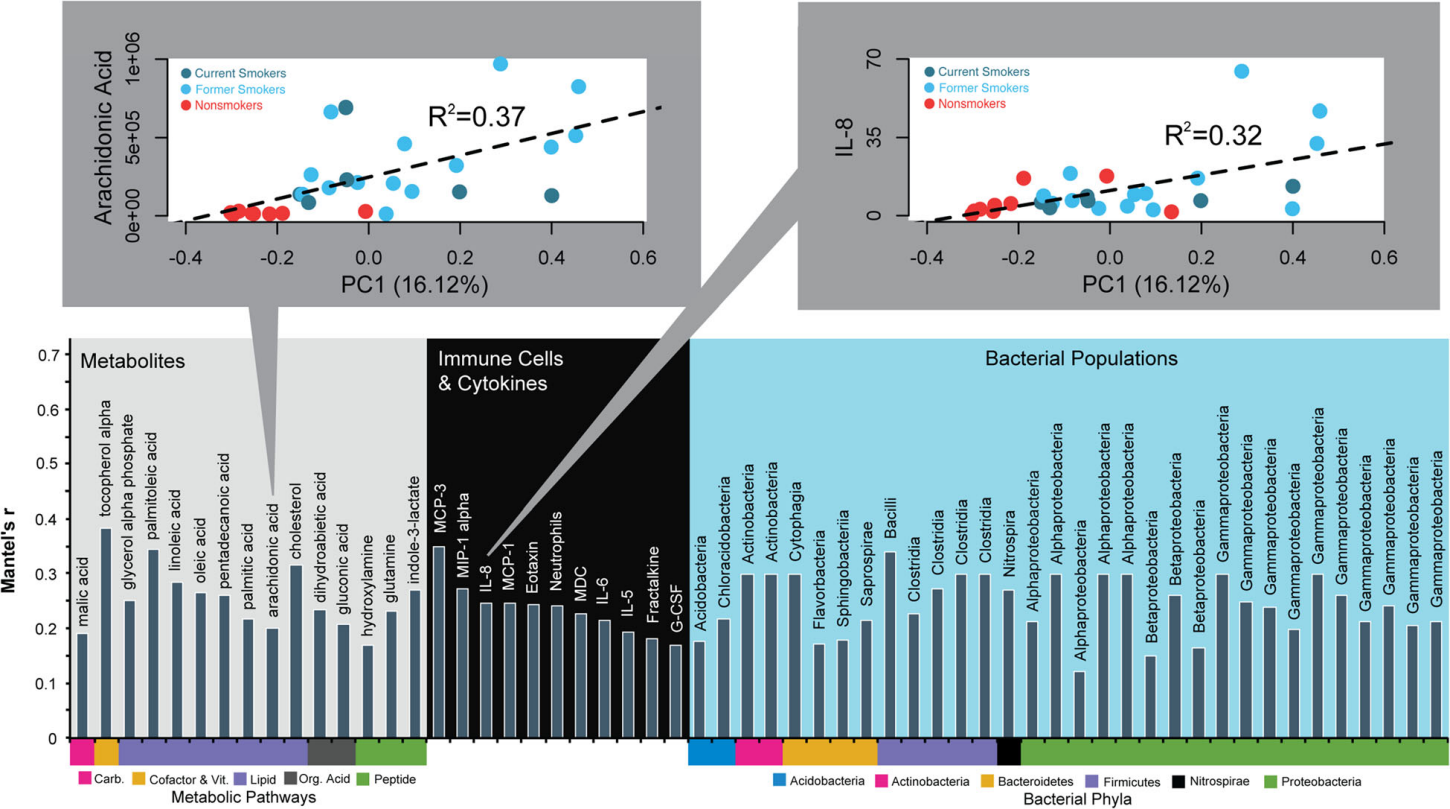
Linkage of Viral Community Changes with the Lung Microenvironment. (bottom) Metabolites, immune cells and cytokines, and bacterial populations with significant correlations (Mantel’s test; *r* > 0.15; *p* < 0.05) to the Bray-Curtis distances between different viral communities. Of the metabolites, immune cells and cytokines, arachidonic acid (top left) and IL-8 (top right) had the highest association (*r*^*2*^ > 0.3) with separation of viral communities based on Bray-Curtis dissimilarity represented by PC1

Understanding how viruses and the pulmonary environment impact each other is important for determining the impact of viruses in the lung. We first evaluated what metabolites, immune cells, cytokines, or bacterial populations might be linked to changes in viral community structure. In total, 15 different metabolites, 11 immune cells and cytokines, and 32 different bacterial populations (Fig. 5) correlated with viral community dissimilarity distances (Mantel’s test, *p* < 0.05, Mantel’s *r* > 0.2). Interestingly, 56% of the bacterial populations correlated with the smoker virome were Proteobacteria, further supporting the role of Proteobacteria and their phages in alterations of host-associated ecosystems [50]. Out of the 26 metabolites, immune cells, and cytokines, arachidonic acid and IL-8 (Fig. 5 top left and top right, respectively) had the highest association with virus community separation based on dissimilarity (*r*^*2*^ > 0.3), and arachidonic acid and IL-8 levels were highest in smokers. No significant differences in IL-8 or arachidonic acid levels were observed between current and former smokers (Mann-Whitney U-test, IL-8 *p* = 0.48, arachidonic acid *p* = 0.13).

## Discussion

In this first study of the effects of smoking on the lung DNA virome, we found that, in contrast to the lung bacteriome, smoking was associated with significant changes in the lung virome and metabolome. Overall, smokers exhibited a contraction of the lung virome, evidenced by lower numbers of viral populations and altered viral ecology. Virome differences between smokers and nonsmokers remained significant even after accounting for age difference between the groups. We hypothesize this altered viral ecology may drive changes in the BAL metabolome between smokers and nonsmokers. Alternatively, changes in the lung metabolic profiles of smokers may lead to downstream effects on the virome, though we consider this less likely as early metabolic changes would presumably also impact bacterial ecology, a link we failed to identify in this study.

Key to our analyses was the ability to quantitatively identify and enumerate viral populations in the lung. While sequence-based 16S rRNA amplification has enabled the rapid quantitative characterization of bacterial communities within the lung [51], the identification and enumeration of respiratory viruses has been much slower due to the lack of a single universal viral marker gene and the difficulty in obtaining sufficient viral biomass from airway samples to sequence without amplification. As a result, all lung virome studies to date have used multiple displacement amplification (MDA) to increase viral DNA yield [14–17]. While this amplification step is useful for amplifying single-stranded DNA viruses, it has both systematic and stochastic biases and results in a non-quantitative representation of community members that varies as much as 10,000-fold from the original [52].

Environmental samples often have low biomass and, as a result, low input DNA, especially in aquatic environments. As a result, most research on producing quantitative viral metagenomes has been done with marine samples, which has shown that samples with as low as 100 femtograms of starting DNA are quantitative if MDA is not used [28, 53–55]. Our lung metagenomes were produced using the DNA-to-sequence pipeline used to produce quantitative marine viromes.

It is important to note that in other systems, reduced microbial diversity is associated with dysbiosis [56]. In the lungs of smokers, such dysbiosis might lead to COPD progression. Previous studies demonstrated differences in the bacteriome of patients with advanced COPD compared to healthy controls [7, 13], however no differences were observed between healthy smokers and nonsmokers [12] suggesting that bacterial dysbiosis may not be responsible for COPD disease progression. In contrast, we found that viral diversity was significantly lower in the lungs of healthy smokers, and this viral dysbiosis was associated almost exclusively with changes in phage ecology. We propose that smoking leads to early effects on the lung virome, and specifically the phageome, which may influence and drive later changes in the bacteriome during progression to COPD. It remains to be determined whether microbial changes lead to disease progression or whether disease progression provides the niche for alterations in the lung microbiome. Well-controlled, longitudinal studies are needed to address this important question.

In the gut, alterations in the number and composition of Proteobacteria is hypothesized to be a signature of dysbiosis and disease [50]. Our corollary finding of associations between two Proteobacterial phages and metabolic changes in smokers parallels these gut findings. Given that Proteobacteria changes were not associated with metabolic differences, we hypothesize that increased numbers of Proteobacteria phages may alter metabolic output within their bacterial hosts during infection.

Previously, we described the presence of bacterial pneumotypes in the lungs of healthy volunteers, thought to be related to the degree of silent aspiration of supraglottic taxa. Using these same specimens, we failed to identify unique viral pneumotypes. Nonetheless, the presence of rare viruses such as *Spiroplasma* phage and human herpesvirus 8, appear to enable colonization by new, closely related common virus types and, thus, may be important for establishing viral pneumotypes (Additional file 4: Figure S3) as has been proposed for bacteria [57, 58]. Analyses of more lung viromes are necessary, however, to clarify the existence of, or lack thereof, viral pneumotypes.

Consistent with prior studies [14, 16–18], the vast majority of viruses identified in our lower airway samples were phages. Nonsmoker viromes were enriched with *Lactobacillus* and *Gardnerella* phages while smoker viromes were enriched with *Prevotella* phages. Prior in vitro work has suggested that a byproduct of cigarette smoke induces *Lactobacillus* phages [59]. However, there are about 4000 compounds in cigarette smoke [60], some of which may induce phage while others may suppress phage, though research in this area is lacking. In our study, the majority of smokers were former smokers and therefore, not recently exposed to cigarette smoke. Additionally, we observed an increased relative abundance of *Lactobacillus* phages in the context of the entire DNA virome of nonsmokers. It is possible that bacteria, phages, or host factors may influence phage induction in the lung microenvironment, as previously demonstrated in co-culture studies of lysogenic bacteria and human epithelial cells [61], factors difficult to model with an ex vivo experiment.

Interestingly, we did not observe crAssphage, a virus found ubiquitously in the human gut and vagina and on the skin [62], in our airway samples, nor did we identify single-stranded DNA anelloviruses. In fact, in our cohort of healthy smokers and nonsmokers, we identified very few eukaryotic DNA viruses in total. The absence of crAssphage may be niche-specific, as it also was not identified in other lung virome studies [14–16]. The absence of anelloviruses in our study may be related to the healthy status of our subjects or to differences in sample preparation and sequence analysis compared to other studies. Anelloviruses have primarily been identified in immunocompromised subjects (lung transplant, HIV or deceased organ donors) using MDA-amplified viromes [14, 17].

We did, however, identify high abundances of *Propionibacterium* phage across all 30 lung BAL samples. Notably, *Propionibacterium spp.* bacteria were previously noted in these samples when 16S rRNA gene sequencing was performed with 454 sequencing of the V1-V2 region [29], but not with Illumina MiSeq sequencing of the V4 region [30], indicating that bacteriome comparisons between studies sequencing different regions of the 16S rRNA gene should be made with caution. While the V4 region is excellent at amplifying bacterial and archaeal 16S rRNA genes [32, 33], it has been shown to be less specific for *Propionibacterium spp.* [63]. Our virome data is consistent with the 454 sequencing of V1-V2 [29] which linked *Propionibacterium spp.* to the “background predominant taxa” bacterial pneumotype as suggested by other studies [49]. Due to the low biomass nature of the lower airways and factors associated with BAL collection, the presence of background taxa in these types of samples is inevitable. However, *Propionibacterium spp.* bacteria have been identified in diseased lungs of subjects with bronchiectasis [64] and sarcoidosis [65] as well as in metagenomic studies of lung tissue and extracellular vesicles [9, 66, 67]. In healthy lungs, the data on *Propionibacterium spp.* bacteria in BAL is conflicting [12, 29, 30, 68]. If *Propionibacterium* phage, like *Propionibacterium spp.* bacteria, represent background, it is important to note that these sequences were found in all samples and were not associated with separation of the virome between smokers and nonsmokers.

We note that changes in phageome composition were not reflected in bacteriome changes. There are several potential explanations for this phenomenon. First, it is impossible to know if the viral nucleic acid and bacterial 16S rRNA genes being sequenced represent live or dead microorganisms. Second, viral reference databases, in general, lack robustness, increasing the challenge of properly aligning and assigning taxonomy to short stretches of viral nucleic acid. To improve the likelihood of identifying viral taxa, we combined multiple viral reference databases into a single, custom database. However, the compositional nature of the relative abundance data will be highly impacted by gaps in the reference database used for annotation. Third, phage-bacteria networks are unique to individuals, vary across body sites and are impacted by environmental factors as recently shown in a network-based analytical model by Hannigan et al. [69]. Therefore, it will be important to continue to consider not only the composition of the microbiome (bacteriome, virome, mycobiome), but also the dynamic interactions between those constituents and with the surrounding environment in future studies.

It is still unclear why some smokers progress to COPD while others remain unaffected, though there is evidence that byproducts of lipoxygenation of arachidonic acid, leukotrienes and lipoxins are important for COPD pathogenesis [70]. Recent studies have also implicated IL-8 as an important potential marker of COPD pathogenesis [71, 72]. Interestingly, of all metabolites and cytokines studied, we observed the strongest association between arachidonic acid and IL-8 and changes in the smoker lung virome. Thus, monitoring specific phage groups or the whole viral community could be important for predicting trends in arachidonic acid and IL-8 and the progression of the smoker lung to COPD. Whether this is a direct interaction or not remains to be determined, but these observations provide a novel pathway of exploration for future studies.

There are several limitations to our study. Statistical power was low in our analyses due to a relatively small sample size. However, due to the invasiveness of the lower airway sampling and cost restraints of our multi-omic approach, particularly in regards to high-throughput next generation sequencing of the virome, we were limited to a cohort of 30 subjects. Nonetheless, our cohort size is in line with current gut virome studies, which do not require an invasive procedure for sample collection. In total, there are 20 gut virome studies with unique datasets [40, 73–91]. Of these studies, the mean number of participants is 35 and the median 20. While smaller than recent lung bacteriome studies, this is the largest study to date to analyze the combined DNA virome, bacteriome and metabolome of BAL fluid. A larger cohort would allow for investigation of the potential role of other important covariates, such as gender, ethnicity, and age, on the lower airway virome. Our study was a cross-sectional analysis of the lower airway microenvironment in smokers and nonsmokers and does not allow for the analysis of trends over time nor the characterization of microbiome changes in relation to COPD progression. Indeed, the lower FEV_1_/FVC ratio observed among smokers may be related to early inflammatory airway dysfunction present at a stage where smokers do not meet COPD criteria [72, 92, 93]. Future longitudinal studies are greatly needed to evaluate whether changes in the lower airway virome have an impact on chronic inflammatory airway dysfunction among smokers. We were also limited by availability of historical specimens as we did not have access to matched oral rinse or pre-bronchoscopy saline control samples of sufficient quantity for shotgun sequencing, thereby precluding characterization of the supraglottic or saline virome. Finally, due to technical constraints, we assessed the acellular BAL DNA virome. Shotgun metagenomics sequences all nucleic acid in a sample, and despite the use of acellular BAL to reduce human genomic contamination, the virome sequence space made up only a tiny fraction of all sequences. Further, in low biomass samples, even small increases in host genomic material will quickly swamp low viral signal. Technical advances in BAL virome purification or enrichment, removal of contaminating host and bacterial nucleic acid, and deeper, more affordable sequencing technologies should be a focus moving forward, thereby allowing more detailed analysis of the lung virome.

## Conclusions

In summary, our findings provide a foundational glimpse into the ecological interplay between viruses, bacteria, metabolites, and immune cells that likely impact the lung microenvironment and ultimately, perhaps, progression from smoking to COPD. We show that, in contrast to the lung bacteriome, the DNA viromes and metabolomes of smokers and nonsmokers are significantly different. We hypothesize that changes in the metabolic output of Proteobacteria in the lungs driven by their phages could potentially be a biomarker for the smoker metabolic disease state. Further, while we cannot disentangle whether arachidonic acid and IL-8 cause alterations in the lung virome or if virome changes cause increases in arachidonic acid and IL-8, these findings suggest that monitoring the lung virome of smokers may be important for assessing the “tipping point” in transitioning from a healthy lung environment to COPD.

## Additional files

Additional file 1: **Table S1.** Virome library read counts. (DOCX 14 kb)

Additional file 2: **Figure S1.** Pie charts of host composition of all bacteriophages. **(A)** Relative distribution of bacteriophage host phyla. **(B-D)** Composition of bacteriophage host genera within the Proteobacteria, Firmicutes, and Actinobacteria host phyla, respectively. (DOCX 911 kb)

Additional file 3: **Figure S2.** Viral community composition of phage by host genera across all virome (overall) and in smokers and nonsmokers. (DOCX 35 kb)

Additional file 4: **Figure S3.** Viral pneumotype analysis using SPIEC-EASI to examine ecological associations based on abundance profiles. (DOCX 60 kb)

Additional file 5: **Figure S4.** Venn diagram of the number of viral populations unique to and shared between smokers and nonsmokers. (DOCX 31 kb)

Additional file 6: **Figure S5.** Comparison of background saline of smokers and nonsmokers. **(A)** PCoA of 16S rRNA gene sequencing data from pre-bronchoscopy control saline samples. **(B)** Heatmap of 16S rRNA OTU abundances (columns) with hierarchical clustering of smoker and nonsmoker pre-bronchoscopy control saline samples (rows). (DOCX 101 kb)

## Abbreviations

BAL: Bronchoalveolar lavage
BEF: Bivariate ellipse fitting
BPT: Background predominant taxa
COPD: Chronic obstructive pulmonary disease
DLCO: Diffusing capacity of the lungs for carbon monoxide
DNA: Deoxyribonucleic acid
FEV1: Forced expiratory volume in 1 s
FRC: Functional residual capacity
FVC: Forced vital capacity
HIV: Human immunodeficiency virus
IL-8: Interleukin 8
PCoA: Principal coordinates analyses
RV: Residual volume
SPT: Supraglottic predominant taxa
TLC: Total lung capacity
