# Supplementary material for: Smoking is associated with quantifiable differences in the human lung DNA virome and metabolome
Source: Respir Res. 2018 Sep 12;19:174. doi: 10.1186/s12931-018-0878-9 (PMC6136173; doi:10.1186/s12931-018-0878-9)
Supplement: Supplementary file 1 — Table S1. Virome library read counts. (DOCX 14 kb) [file 12931_2018_878_MOESM1_ESM.docx]

**Additional file 1: Table S1 Virome library read counts.**

| **Virome Library** | **Type** | **Total Number of Reads** | **Number of Reads Following Removal of Human Sequences** | **Number of Reads Mapping to Viral Databases** |
| --- | --- | --- | --- | --- |
| m038 | Smoker | 2,063,504 | 987,026 | 323 |
| m039 | Smoker | 3,576,930 | 1,556,894 | 400 |
| m040 | Smoker | 2,586,386 | 1,067,199 | 342 |
| m041 | Smoker | 4,363,460 | 1,844,251 | 488 |
| m042 | Smoker | 2,164,832 | 1,014,876 | 402 |
| m043 | Smoker | 3,090,562 | 1,257,069 | 392 |
| m044 | Smoker | 4,882,478 | 2,051,981 | 512 |
| m045 | Smoker | 2,307,222 | 1,103,975 | 321 |
| m046 | Smoker | 5,425,990 | 2,476,372 | 534 |
| m047 | Smoker | 12,098,092 | 5,103,051 | 897 |
| m048 | Smoker | 7,927,680 | 3,506,391 | 1172 |
| m049 | Smoker | 990,206 | 474,629 | 166 |
| m050 | Smoker | 1,339,468 | 616,741 | 182 |
| m051 | Smoker | 2,381,842 | 1,130,476 | 264 |
| m052 | Smoker | 1,537,708 | 737,628 | 211 |
| m053 | Smoker | 974,360 | 466,329 | 138 |
| m054 | Smoker | 1,007,568 | 486,572 | 190 |
| m055 | Smoker | 1,143,876 | 546,661 | 154 |
| m056 | Smoker | 1,073,858 | 518,486 | 156 |
| m057 | Smoker | 1,151,280 | 553,482 | 150 |
| m094 | Nonsmoker | 2,025,414 | 933,090 | 242 |
| m095 | Nonsmoker | 938,726 | 427,697 | 175 |
| m096 | Nonsmoker | 1,409,104 | 639,116 | 237 |
| m097 | Nonsmoker | 1,677,604 | 787,065 | 262 |
| m098 | Nonsmoker | 881,880 | 408,038 | 138 |
| m099 | Nonsmoker | 1,145,938 | 526,052 | 193 |
| m100 | Nonsmoker | 1,212,034 | 552,639 | 240 |
| m101 | Nonsmoker | 3,662,680 | 1,502,270 | 358 |
| m102 | Nonsmoker | 1,148,578 | 498,096 | 211 |
| m103 | Nonsmoker | 1,695,498 | 793,114 | 280 |
