# Supplementary figures and images for: Smoking is associated with quantifiable differences in the human lung DNA virome and metabolome
Source: Respir Res. 2018 Sep 12;19:174. doi: 10.1186/s12931-018-0878-9 (PMC6136173; doi:10.1186/s12931-018-0878-9)

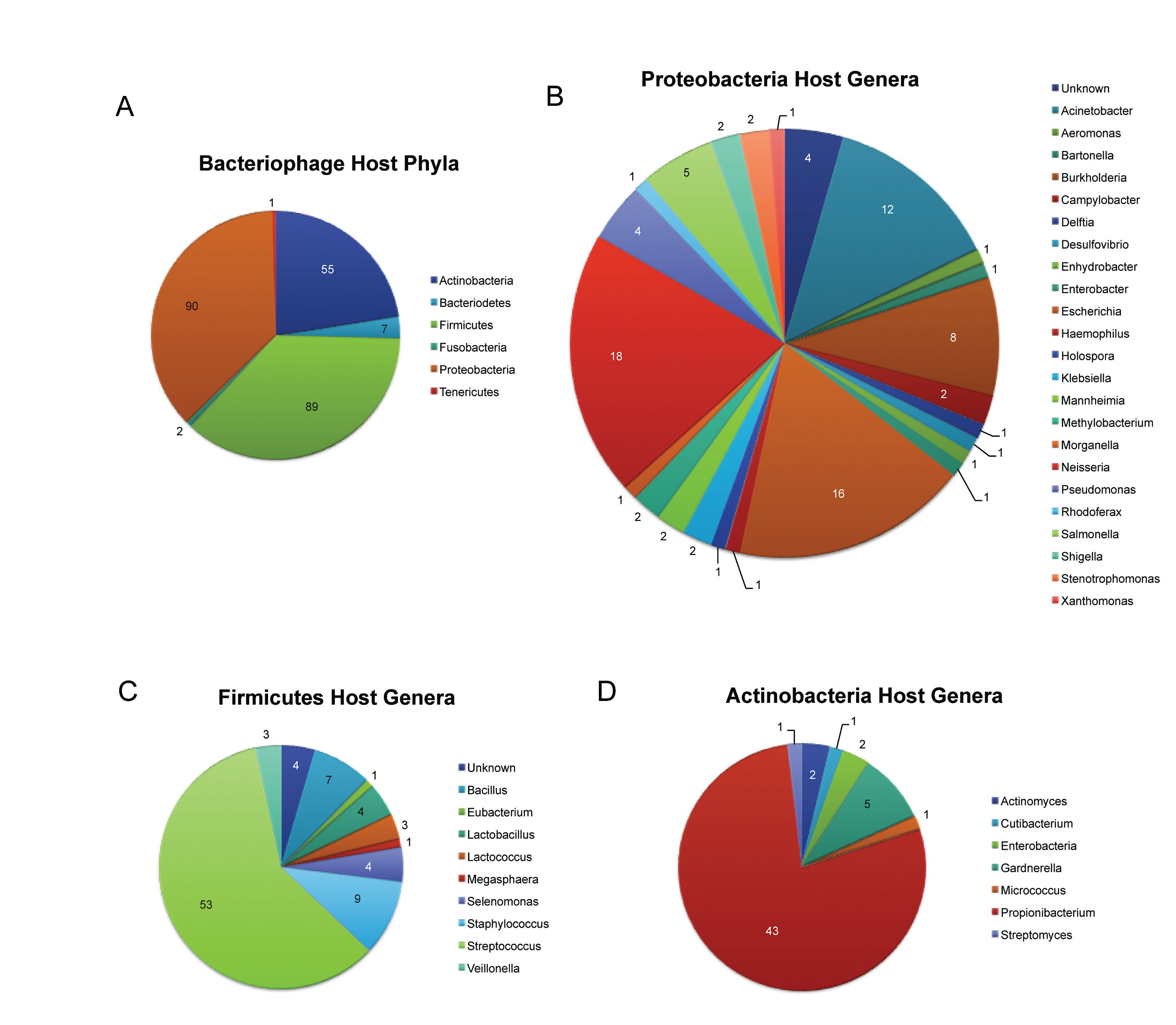

Supplement: Supplementary file 2 — Figure S1. Pie charts of host composition of all bacteriophages. (A) Relative distribution of bacteriophage host phyla. (B-D) Composition of bacteriophage host genera within the Proteobacteria, Firmicutes, and Actinobacteria host phyla, respectively. (DOCX 911 kb) [file 12931_2018_878_MOESM2_ESM.docx]

**
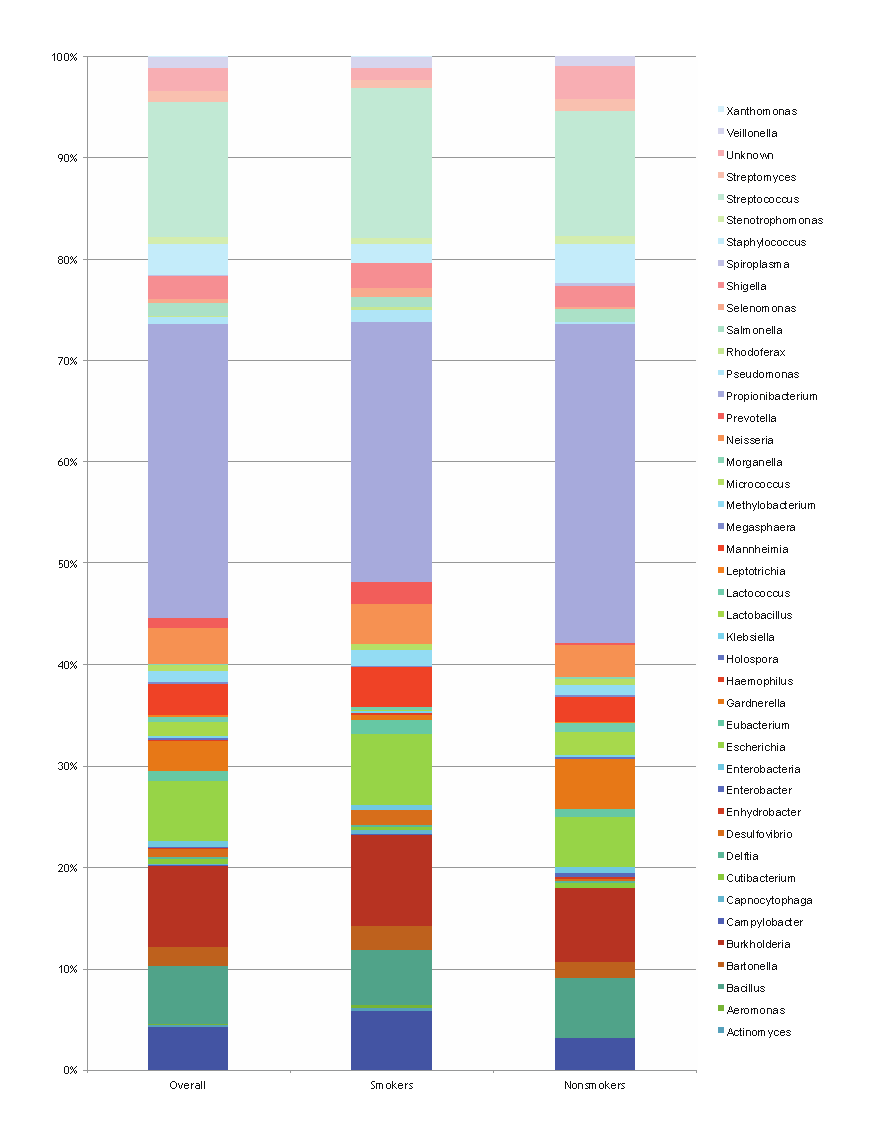
**

Supplement: Supplementary file 3 — Figure S2. Viral community composition of phage by host genera across all virome (overall) and in smokers and nonsmokers. (DOCX 35 kb) [file 12931_2018_878_MOESM3_ESM.docx]

**
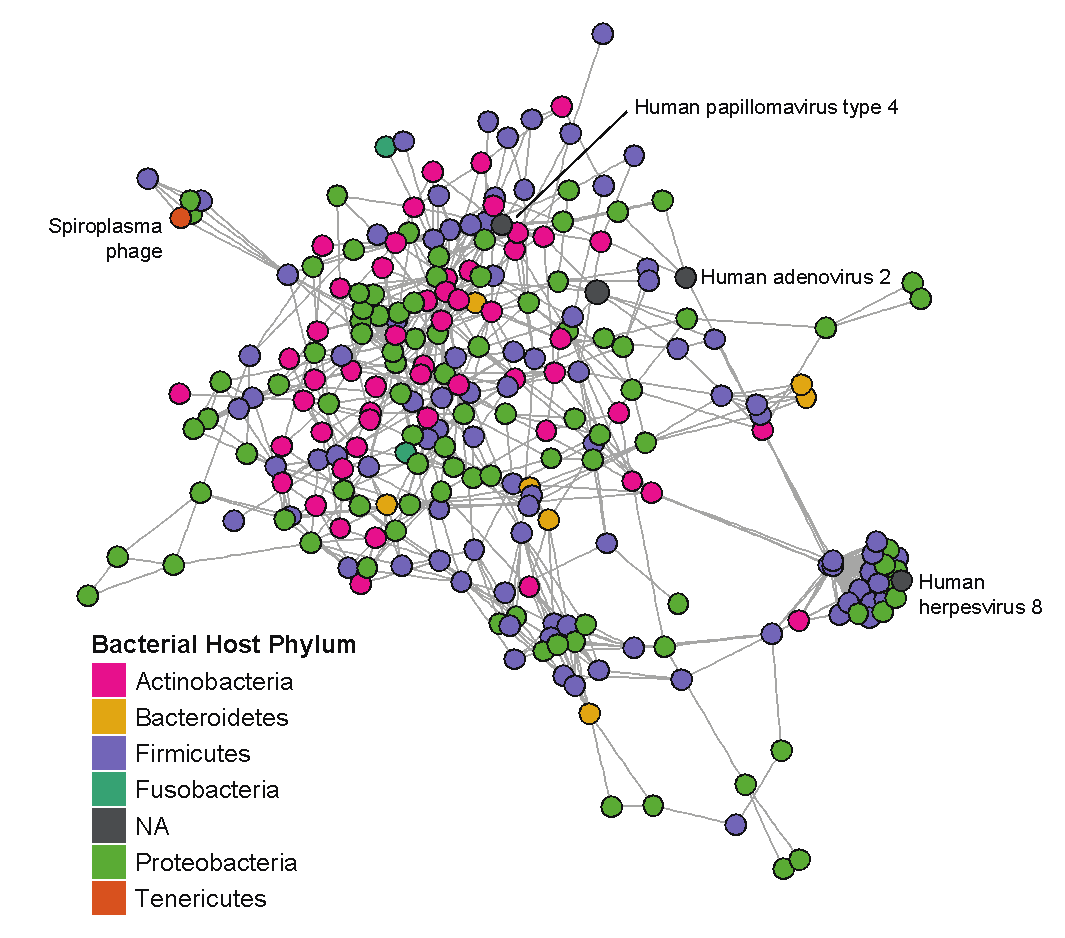
**

Supplement: Supplementary file 4 — Figure S3. Viral pneumotype analysis using SPIEC-EASI to examine ecological associations based on abundance profiles. (DOCX 60 kb) [file 12931_2018_878_MOESM4_ESM.docx]

**
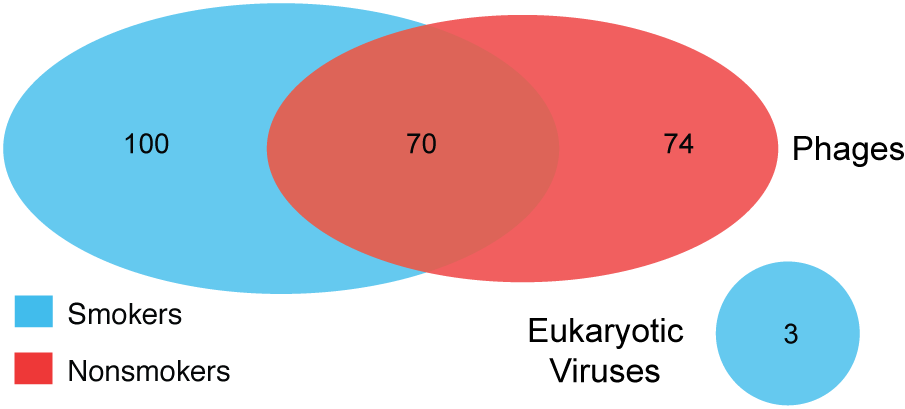
**

Supplement: Supplementary file 5 — Figure S4. Venn diagram of the number of viral populations unique to and shared between smokers and nonsmokers. (DOCX 31 kb) [file 12931_2018_878_MOESM5_ESM.docx]

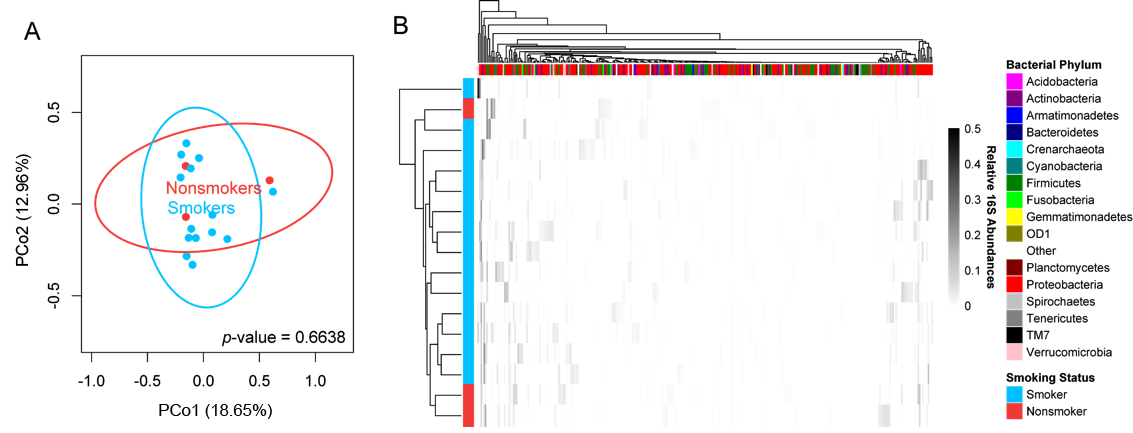

Supplement: Supplementary file 6 — Figure S5. Comparison of background saline of smokers and nonsmokers. (A) PCoA of 16S rRNA gene sequencing data from pre-bronchoscopy control saline samples. (B) Heatmap of 16S rRNA OTU abundances (columns) with hierarchical clustering of smoker and nonsmoker pre-bronchoscopy control saline samples (rows). (DOCX 101 kb) [file 12931_2018_878_MOESM6_ESM.docx]
